# Musical instrumental reading affects middle cerebral blood flow and cognitive function

**DOI:** 10.3389/fphys.2022.966969

**Published:** 2022-08-29

**Authors:** Ai Kawasaki, Naoyuki Hayashi

**Affiliations:** ^1^ Department of Social and Human Sciences, Tokyo Institute of Technology, Tokyo, Japan; ^2^ Institute for Liberal Arts, Tokyo Institute of Technology, Tokyo, Japan; ^3^ Faculty of Sport Sciences, Waseda University, Saitama, Japan

**Keywords:** Cognitive function, cerebral artery, playing an instrument, reading music, transcranial Doppler flowmetry

## Abstract

Playing a musical instrument includes reading music scores, playing, and listening in parallel. It is unclear which of these activities are responsible for an increase in cerebral blood flow. We investigated the factors increasing middle cerebral artery velocity (MCAv) during musical performance, and examined whether playing and reading music affects cognitive function. Seventeen musicians played an instrument with reading music, played music from memory, and read music scores in a randomized order, for 10 min each. The MCAv was continuously recorded from 5 min before to 10 min after the performance. A Stroop test was performed before and after performance. The MCAv increased significantly with reading music, playing from memory, and reading music. Stroop test scores increased significantly after music reading. These findings suggest that both music reading and playing an instrument are involved in the increase in MCAv during music performance. Cognitive function was transiently improved by playing musical instruments.

## 1 Introduction

Cerebral blood flow (CBF) increases during music playing. Functional magnetic resonance imaging (fMRI) and positron-emission topography studies have shown that many areas of the brain are activated while a person plays a musical instrument. Musicians showed activation in premotor, primary motor, and supplementary motor areas during both performance and auditory tasks (Hund-Georgiadis and Yves Von Cramon, 1999; Meister et al., 2004; Baumann et al., 2005). Scale playing and concerto performance activates primary motor cortex, corresponding somatosensory cortex, inferior parietal cortex, supplementary motor cortex, motor cortex, bilateral superior and middle temporal cortices, right thalamus, and both anterior and posterior cerebellum (Parsons et al., 2005). By using transcranial Doppler techniques, we reported an increase in middle cerebral artery blood flow velocity (MCAv) by 3–9% when subjects played a musical instrument (Kawasaki and Hayashi, 2022). This suggests absolute increases in whole and/or regional cerebral blood volume and/or flow during music playing.

Musical performance requires simultaneous coordination of sound by reading a music score, playing an instrument, listening, and feedback from the sound generated. This series of generating music reflects activation of the cerebral cortex. Playing with the right hand activates the left motor cortex, the right cerebellum corresponding to motor representations of the right hand, and the left premotor area, while listening to musical scales activates the bilateral secondary auditory cortex and the superior temporal gyrus in the left hemisphere. In addition, reading musical notation activates bilateral extrastriate visual areas (Sergent et al., 1992). Humans commonly perceive musical features such as chords, harmonies, sensations, and rhythms when reading music, playing, and listening to music. This perception takes place in a network that includes the inferior anterior medial and prefrontal cortices, the premotor cortex, the anterior and posterior parts of the superior temporal gyrus, and the inferior parietal lobe (Janata et al., 2002; Patel, 2003). It is unclear which of the following factors is responsible for the increase in CBF associated with performance: playing an instrument, listening to music, and reading music scores.

Physical activity transiently improves cognitive function (Oken et al., 2006; Erickson et al., 2019). Playing an instrument is one type of physical activity. It requires complex muscle activities whereby it recruits smaller muscle mass with lighter intensity than general physical activities. Studies analyzing the movements of the fingers when playing the piano (Rahman et al., 2011) and the coordinated movements of the violin and bow (Maestre et al., 2007) show that playing requires complex muscular activities. Musical activities such as playing instruments, listening to music, and composing stimulate a variety of cognitive functions. Listening to music improves cognitive function in the domains of verbal memory and concentration during early recovery from middle cerebral artery stroke (Särkämö et al., 2008). Older, active musicians and non-musicians differ in word recognition, nonverbal memory recall, visuomotor speed, visuomotor continuity, and cognitive flexibility function (Hanna-Pladdy and MacKay, 2011). Thus, musical activity may transiently increase cognitive function as well as physical activity.

The primary objective of this study was to examine the role of reading score in the increase in CBF associated with playing an instrument at different tempos. To accomplish this aim, we compared the responses in CBF among playing and reading music at fast and slow tempos, and playing without looking at the score. The secondary objective of the study aimed to examine whether playing music increases cognitive function. We hypothesized that an assumed increase in CBF associated with playing results in improvement in cognitive function as shown in physical activity.

## 2 Materials and methods

### 2.1 Participants

Seventeen healthy musicians (eight males and nine females; six violinists and eleven pianists, aged 26 ± 8.9 years) participated in the study. All have received classical music training. The participants had been regularly playing their instruments for >6 years. The subjects had no history of autonomic nervous system disorders or heart disease and had not smoked or taken any medication.

All participants provided written consent to participate in this study after receiving an explanation regarding its purpose and their involvement. This study was approved by the Human Subjects Research Ethics Review Committee of Tokyo Institute of Technology (2017098).

### 2.2 Experimental protocol

Participants were asked to refrain from consuming caffeine and performing strenuous exercise for 6 h before the experiment, and from eating for 2 h before the experiment. Each participant played Pachelbel’s Canon, designated as the test piece, for 10 min at a slow tempo of eighth note = 60 and a fast tempo of eighth note = 80 (PL60 and PL80). They were asked to play the music with reading the music score. They also read the music scores at the same tempo without playing the instrument for 10 min (RE60 and RE80). The participants were asked to read the music score silently and they were instructed not to move and not to imitate playing the musical instrument. The tempo was provided on a metronome before the performance and instructed to be maintained until the end of the performance. To exclude the effects of visual processing during playing, 13 participants (five violinists and eight pianists) played from memory, without looking at music for 10 min (ME80). The order of the five trials was randomized for each participant, and there was a sufficient resting period between the trials. All trials were done without listening to recording nor to playing of others.

### 2.3 Measurements

The MCAv was measured by using a transcranial ultrasonic Doppler flowmetry (WAKI, Atys Medical, St-Genis-Leval, France), whose probe was applied to the left temporal region *via* a headband. Blood pressure was recorded using an automatic sphygmomanometer (UA-704, A&D, Tokyo Japan).

Stroop’s color word test (SCWT) was used as a cognitive test; this is a neuropsychological test focusing on the task of color naming and word interference, developed by Stroop (Scarpina and Tagini, 2017). The participants performed a 1-min SCWT before, and immediately and 10 minutes after the music trial. This test involved displaying a word in incongruent colors; blue, red, green, yellow, orange, white, brown, pink, purple, and black on a smartphone monitor. The participant was instructed to indicate the color in which the word was written on the monitor. Prior to the test, each participant received instructions on how to perform the test and practiced until they became used to the testing. The number of clicks, correct answers, and errors were counted using the smartphone application SCWT color (Tekxudus; https://apkfab.com/developer/Tekxudus). The total score was calculated by subtracting the number of errors from the number of correct answers.

### 2.4 Data analysis

Data were expressed as mean ± standard deviation. The MCAv for the last minute of each duration at rest, performance, and recovery were used for analysis. Two-way analysis of variance was used to examine the effects of time and trial. The effects of instrumentation between PL80 and RE80 and PL60 and RE60, and the effects of tempo between PL80 and PL60 and RE80 and RE60 were examined by two-way analysis of variance. T-tests were performed on ME80 and PL80 to examine the effect of reading the music score. When a significant F value was obtained, the variables at rest, playing, and recovery were compared using Dunnett’s post hoc test. The level of statistical significance was set at *p* ≤ 0.05. IBM SPSS Statistics 21.0 (IBM Corp. Armonk, NY, United States) was used for all statistical analyses.

## 3 Results

**FIGURE 1:**
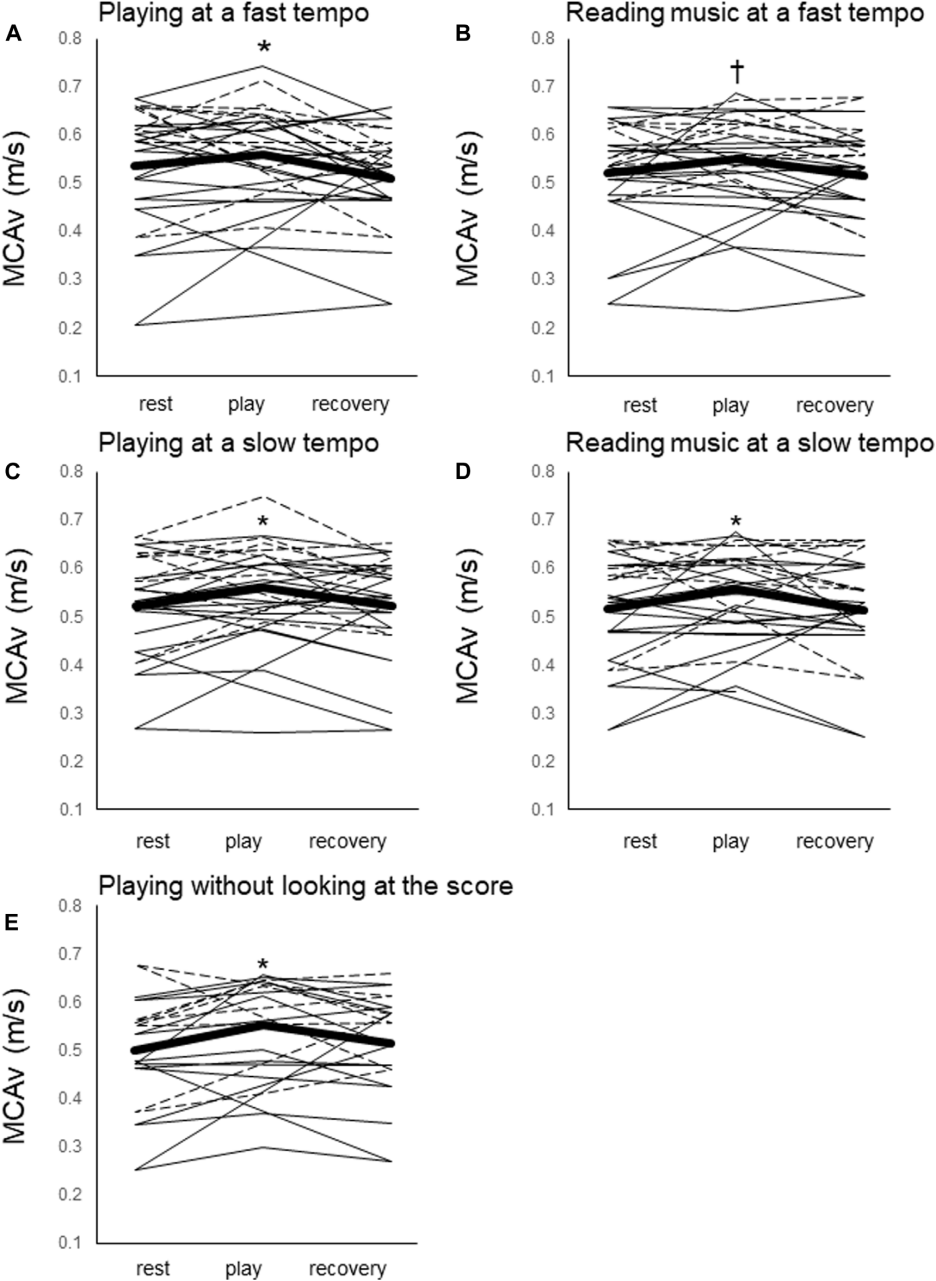
Middle cerebral artery blood flow velocity (MCAv) before, during, and after five trials. **(A)** Playing at a fast tempo (PL80), **(B)** reading music at a fast tempo (RE80), **(C)** playing at a slow tempo (PL60), **(D)** reading music at a slow tempo (RE60), and **(E)** playing without looking at the score (ME). Mean values for all participants (thick black line) and individual values for piano players (thin black line) and violin players (dotted line) are shown. *: *p* < 0.05 vs. resting. †: *p* < 0.07 vs. resting.

MCAv at rest was similar among trials (Figure 1). An effect of time on MCAv was observed. MCAv increased significantly by 5%, 7%, 6%, 9% and 12% from baseline values in the PL80 (0.53 ± 0.1 m/s at rest vs. 0.56 ± 0.1 m/s during play, F = 5.95, *p* = 0.03), PL60 (0.52 ± 0.1 m/s at rest vs. 0.56 ± 0.1 m/s during play, F = 6.73, *p* = 0.004), RE80 (0.52 ± 0.1 m/s at rest vs. 0.56 ± 0.1 m/s during play, F = 4.69, *p* = 0.07), RE60 (0.51 ± 0.1 m/s at rest vs. 0.56 ± 0.1 m/s during play, F = 7.14, *p* = 0.02), and ME80 (0.49 ± 0.1 m/s at rest vs. 0.56 ± 0.1, F = 7.64, *p* = 0.01) trials, respectively. These significant increases were not seen in recovery duration.

There were no significant differences in the increase in MCAv among trial. The effect of playing an instrument was not shown between PL80 and RE80, PL60 and RE60 (F = 0.26, *p* = 0.41). The effect of tempo was not shown between PL80 and PL60 or RE80 and RE60 (F = 0, 68, *p* = 0.609). No significant effect of looking at the score was also found between ME80 and PL80 (t = 1.70, *p* = 0.1).

The resting MBP values were similar at rest. No significant effects in trials and time were found in MBP (Table 1).

**TABLE 1:** Mean blood pressure (MBP) before, during, and after trials. **(A)** Playing at a fast tempo (PL80), **(B)** reading music at a fast tempo (RE80), **(C)** playing at a slow tempo (PL60), **(D)** reading music at a slow tempo (RE60), and **(E)** playing without looking at the score (ME). Data are means ± SD and *p* value for comparing rest vs. during play. No significant differences were shown.

	MBP (mmHg)			
	Rest	Play	Recovery	*p* value
A.PL80	86±14	86±12	88±13	1.0
B.RE80	87±14	86±14	86±13	1.0
C.PL60	87±14	86±13	88±16	1.0
D.RE60	88±16	84±12	88±12	0.42
E.ME80	87±11	90±12	86±12	0.23

**FIGURE 2:**
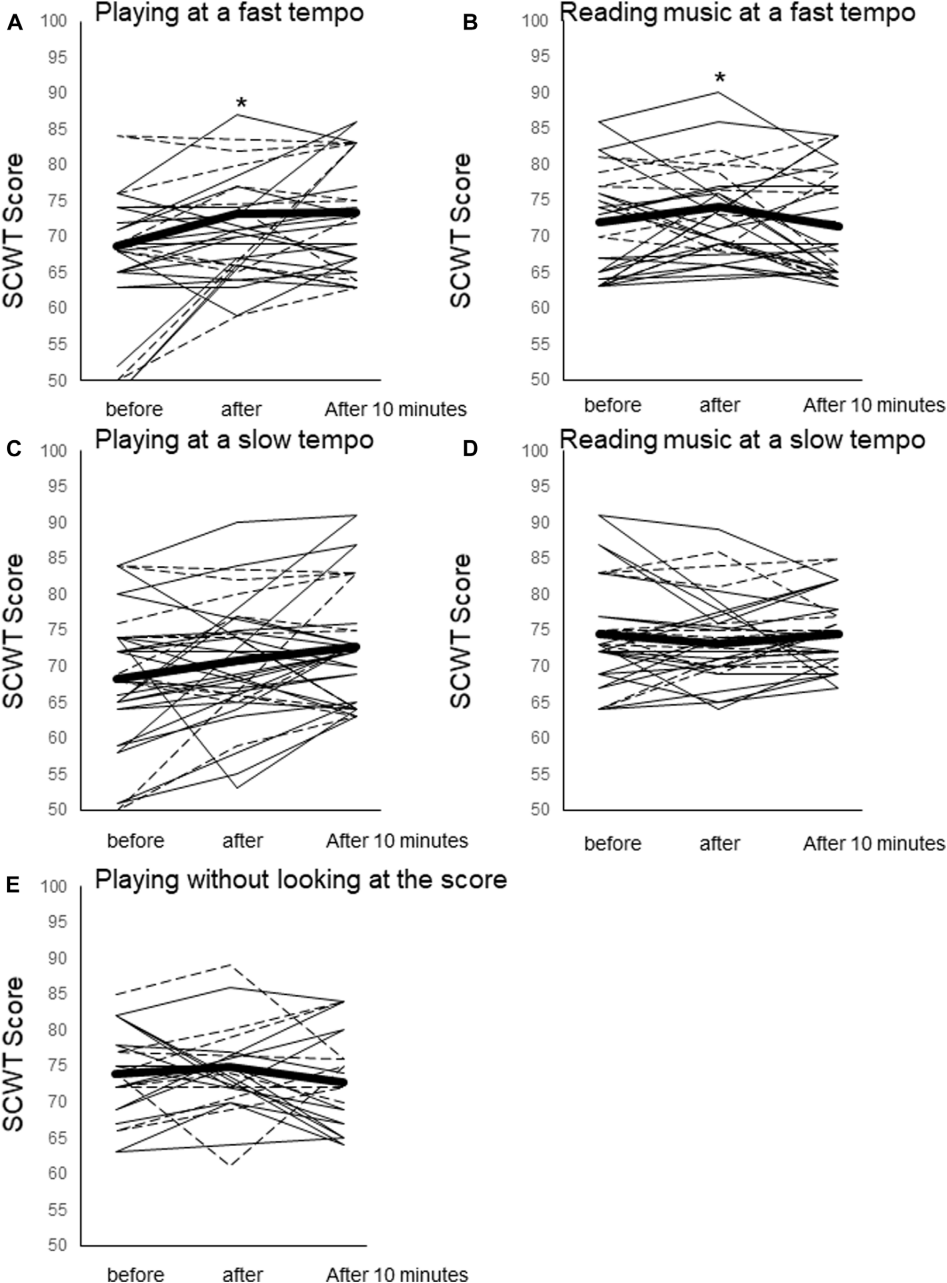
Stroop test (SCWT) scores before, immediately after the trial, and 10 min after the trial. **(A)** Playing at a fast tempo (PL80), **(B)** reading music at a fast tempo (RE80), playing at a slow tempo (PL60), reading music at a slow tempo (RE60), and playing without looking at the music (ME). Mean values for all participants (thick black line) and individual values for piano players (thin black line) and violin players (dotted line) are shown. *: *p* < 0.05 vs. resting. †: *p* < 0.07 vs. resting.

The SCWT score increased significantly from resting immediately after play for PL80 (68 ± 8.6 at rest vs. 73 ± 7.3 after play, F = 5.68, *p* = 0.04) and for RE80 (71 ± 7.3 at rest vs. 74 ± 7.2 after play, F = 3.10, *p* = 0.004), while no significant changes were shown in other trials.; PL60 (70 ± 9.8 at rest vs. 74 ± 10 after play, F = 6.06, *p* = 0.68), RE60 (74 ± 7.4 at rest vs. 73 ± 6.7 after play, F = 0.98, *p* = 0.82) and ME80 (74 ± 6.4 at rest vs. 75 ± 7.0 after play, F = 0.76, *p* = 1.00) (Figure 2).

**FIGURE 3:**
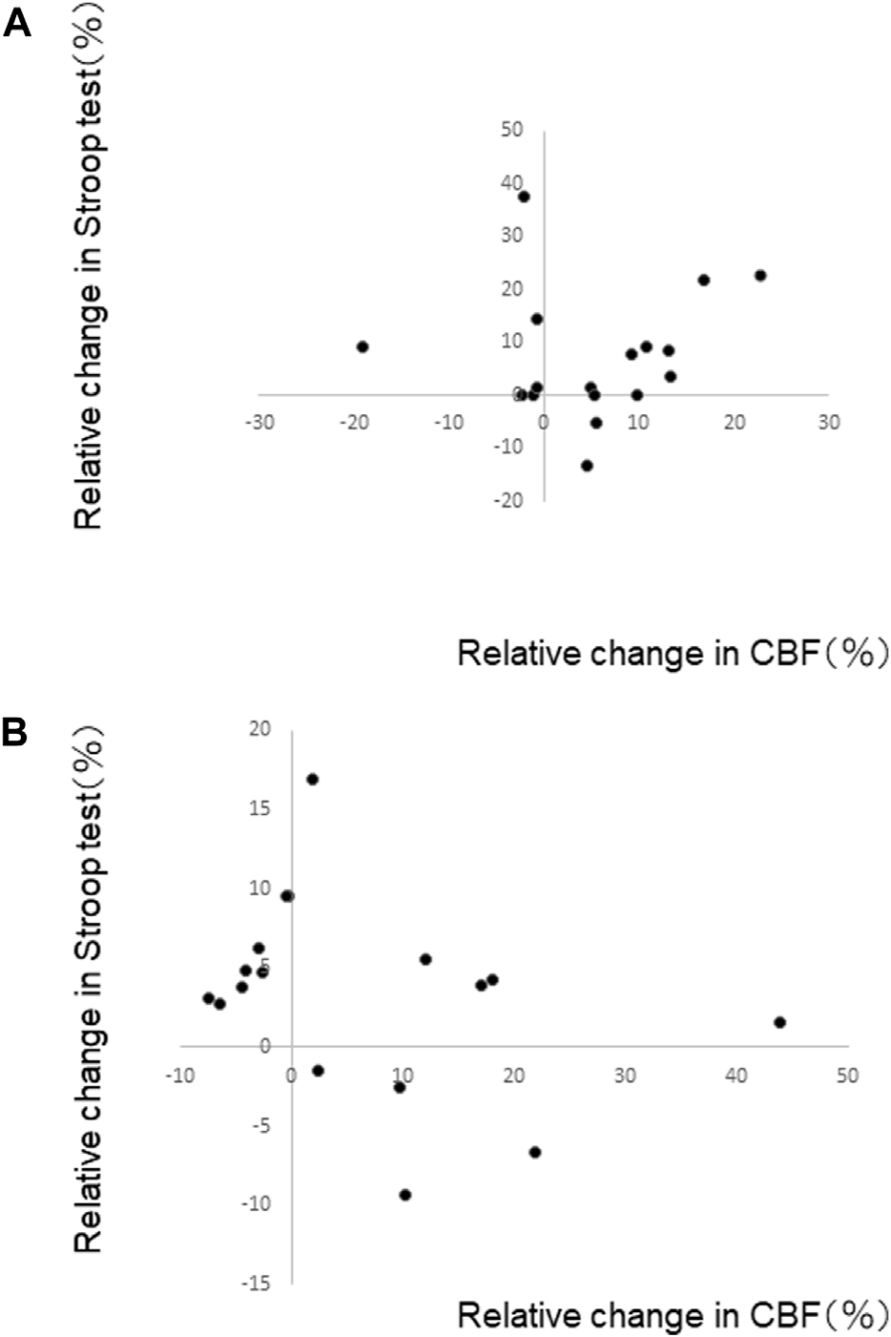
Relative change in Stroop test (SCWT) scores and relative change in middle cerebral artery blood flow velocity (MCAv) for **(A)** playing at a fast tempo (PL80) and **(B)** reading music at a fast tempo (RE80). The MCAv for the last minute of performance and the SCWT immediately after the performance were used. No significant relationship was shown.

We examined a relationship between the relative changes in MCAv and SCWT scores for PL80 and RE80, where we observed increases in both MCAv and SCWT scores. The MCAv for the last minute of performance and the SCWT immediately after the performance were used. There was no significant correlation between the changes in MCAv and SCWT scores; PL80 (r <0.01) and RE80 (r <0.1) (Figure 3).

## 4 Discussion

We examined the effect of playing instruments and reading music scores separately on MCAv and SCWT score. The results showed that playing an instrument and reading music both significantly increased MCAv by a similar degree, suggesting that playing an instrument, playing from memory and reading music are involved in the increased MCAv observed during playing music (Kawasaki and Hayashi 2022). The increase in PL80 and PL60 corresponded to our previous study reporting the increase in MCAv by 3–9%. The increase in RE60 and ME80 corresponded to the increase during playing instruments, in contrast being smaller than exercise, e.g., roughly 10–20% increase, during dynamic exercise (Sato et al., 2011). The increased MCAv during reading music alone suggests that the increased MCAv is not solely due to muscular activities associated with playing an instrument; this implies that reading music score plays a role. In turn, the similar increase in MCAv during playing without reading music score (ME80) indicates that playing even without a visual task relating to reading music increases MCAv, suggesting a role of recalling, physical activity, and/or adjusting the sound required to play the music. Roles of reading, physical activity, adjusting the sound by feedback on the increase in MCAv are suggested, though we cannot establish or deny each contribution due to the complex contribution of these factors on cerebral blood flow. The significant increase in SCWT score was observed immediately after playing and reading music at faster tempo, partly supporting our hypothesis that playing music increases the SCWT score as well as physical activity.

The increased MCAv during reading music can be explained by the effects of imagination of musical performance and reading music score. An fMRI study has shown that the supplementary motor area and premotor area are activated when pianists, violinists, and cellists imagine playing an instrument (Langheim et al., 2002). Activation of the premotor area has also been observed in both conditions when a pianist is actually playing music and when imagining playing music (Meister et al., 2004). Activations in these areas can related to the increase in MCAv during reading music.

Playing with reading music during playing music also activates variable areas of the brain, e.g., visual areas outside the bilateral striatum, the left posterior parieto-occipital (Sergent et al., 1992), right posterior junction, superior parietal lobule, and intraparietal sulcus (Schön et al., 2002). MCAv increases with score reading probably in order to supply blood to these regions.

The increase in MCAv may be partly due to the brain’s recognition of music through the playing and reading of it. Some brain regions involved in music processing are supplied by the MCA (Ayotte et al., 2000), i.e., premotor and supplementary motor areas, and visual areas. MCA stroke patients have deficits in music cognition (Altenmüller et al., 2009; Särkämö et al., 2009). Furthermore, 35% of patients with a ruptured aneurysm in the MCA have been reported to have impaired music recognition (Ayotte et al., 2000). During playing and reading music, information is processed to recognize music, activating the relevant brain regions. This may result in increased blood flow to the MCA.

The playing tempo did not affect the magnitude of increase in MCAv during playing and reading music. There was no significant difference in MCAv between fast and slow tempo in our study. A study using near-infrared spectroscopy found a greater increase in total hemoglobin at the anterior lobe during finger tapping at maximal effort than finger tapping at 25% of maximal effort (Kuboyama et al., 2004). The different results may be due to differences in the target area of brain and tempo. In our study, blood flow velocity was observed in the MCA, which supplies blood to the dorsolateral, inferior frontal cortex (including Broca’s area), superior temporal gyrus (Wernicke’s area), supramarginal gyrus, supplementary motor area, and premotor area (Bangert et al., 2006), which are the networks activated during musical performance. In the previous study, blood flow velocity in the left primary motor cortex was observed. In our study, the tempo was set at = 60 and 80 eighth notes; for the skilled musicians the tempos in were far less than maximum finger tapping tempo. Thus, the difference in the tempo was so less likely to induce a significant increase in MCAv. The effect of playing at a faster tempo is a subject for future study.

Playing an instrument increases the SCWT score as shown after an acute exercise (Erickson et al.; Oken et al., 2006). The RE trial also showed an increase, suggesting that SCWT scores may increase even without playing an instrument, i.e., physical activity. SCWT scores increased 8% during instrumental performance and 3% during music reading. These slight but significant increases were seen only in the PL80 and RE80 trials. The ME80 trial did not show a significant increase in SCWT score despite the same tempo as PL80 and RE80. Further studies should examine conditions improving cognitive function by music and also should compare the effect of reading music score and reading books in order to examine whether reading music or reading books is a requirement for increasing CBF.

Our study failed to show a possible mechanism increasing MCAv during playing music. We observed no significant relationship between MCAv and SCWT score, while both increased by playing music. It is still unclear what mechanisms improve cognitive function after acute exercise although previous studies have reported such an improvement. Cognitive function improves during acute moderate-intensity exercise (Brisswalter et al., 2002; McMorris and Hale, 2012). It is well known that exercise increases cerebral blood flow and improves cognitive performance at the same time. Nevertheless, these do not seem to relate each other. For example, cognitive performance increased during prolonged exercise despite a decrease in MCAv (Ogoh et al., 2014); it was suggested that the improvement in cognitive performance due to exercise was not simply due to changes in CBF. Our study may support this notion; the increase in SCWT score after playing an instrument improves cognitive function irrespective of the nature of MCAv. Further studies may be required to examine the mechanism, though it is difficult to clearly describe the mechanism simply since the cognitive functions relate complex neural processes.

We have two limitation in the present study. First, the difficulty of the music was not controlled; test music piece could have been easily performed for some subjects having higher performance ability. MCAv could not have responded to a smaller stimulus in these subjects. The difficulty controlling the difficulty of the music is a limitation of the present study, and maybe other studies using music, even though the conditions for the test music piece and duration of experiment were simply controlled. Second, this study cannot be applied to the performance of wind instruments and singing. One of the physiological factors affecting MCAv is the arterial blood carbon dioxide concentration, which can be easily affected by ventilation. In turn, pianists and violinists do not require so much ventilation to perform.

In conclusion, MCAv increases while playing a musical instrument, with or without reading music. This suggests that playing a musical instrument may temporarily improve cognitive function. However, it remains unclear whether the improvement in cognitive function achieved by playing a musical instrument is associated with an increase in CBF.

## Data Availability

The original contributions presented in the study are included in the article/supplementary materials, further inquiries can be directed to the corresponding author.
